# Supplementary material for: Expression of the MSPDBL2 antigen in a discrete subset of Plasmodium falciparum schizonts is regulated by GDV1 but may not be linked to sexual commitment
Source: mBio. 2024 Mar 26;15(5):e03140-23. doi: 10.1128/mbio.03140-23 (PMC11077968; doi:10.1128/mbio.03140-23)
Supplement: Supplemental Figures and Tables — Figs S1-S2; Tables S1-S13. [file mbio.03140-23-s0001.docx]

Supplementary Figures and Tables (Figures S1-S2, Tables S1-S13)


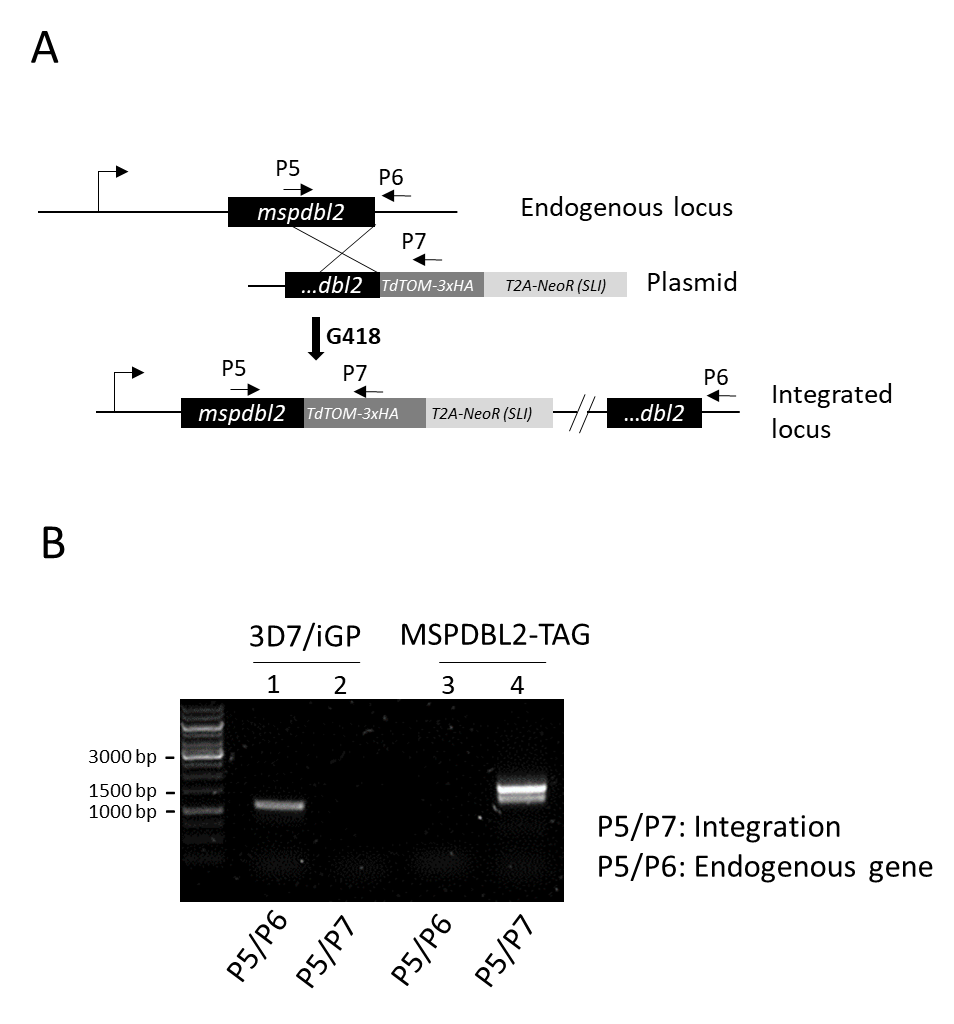


**Figure S1. Generation of a *Plasmodium falciparum* line to express a TdTOM-3xHA tagged version of MSPDBL2 in a majority of schizonts (MSPDBL2-TAG). (A)** Schematic representation of C-terminal tagging of MSPDBL2 with TdTOM-3xHA using the selection-linked integration (SLI-T2A/G418) single homologous recombination strategy. At the endogenous *mspdbl2* locus, the entire *mspdbl2* coding sequence (3D7 allele) is engineered in frame with TdTOM-3xHA and the *t2a* peptide linked to the *neomycin* selection marker (the contiguous engineered DNA sequence is shown in Table S13). Transgenic parasites were selected using G418. The position of the primers used for diagnostic PCR (listed in Table S12) are indicated by arrows. **(B)** Diagnostic PCR of MSPDBL2-TAG (lanes 3-4) compared to the parental 3D7/iGP line (lanes 1-2). Lanes 1 and 3: endogenous (P5-P6). Lanes 2 and 4: 5’integration (P5 and P7).

**
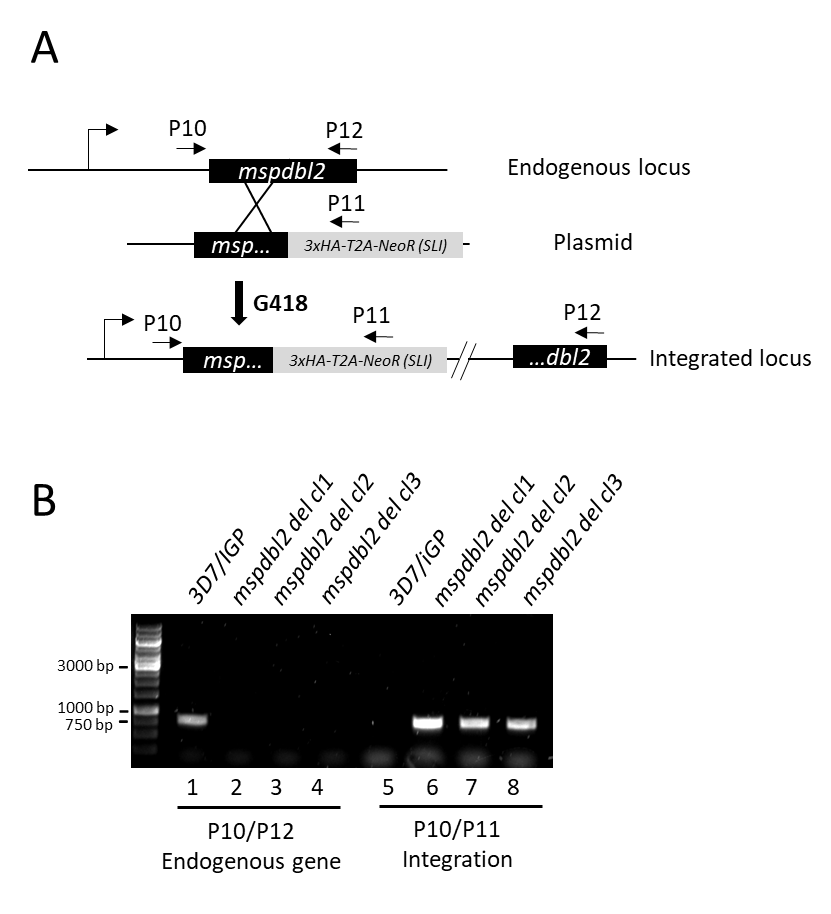
**

**Figure S2. Generation of a *Plasmodium falciparum* line to express a disrupted version of MSPDBL2 in a majority of schizonts (MSPDBL2 DEL lines). (A)** Schematic representation of the *mspdbl2* disruption strategy using selection-linked integration (SLI-T2A/G418) single homologous recombination. At the endogenous *mspdbl2* locus, the truncated version of *mspdbl2* is engineered in frame with and expressed as a 3xHA-tagged fusion containing the *t2a* peptide linked to the *neomycin* selection marker (the contiguous engineered DNA sequence is shown in Table S13). Transgenic parasites were selected using G418. The position of the primers (Table S12) used for diagnostic PCR are indicated by arrows. **(B)** Diagnostic PCR of MSPDBL2 DEL: Lanes 1-4: detection of the endogenous *mspdbl2* (P10-P12). Lanes 5-8: *mspdbl2* gene disruption (P10-P11). Lanes 1 and 5: 3D7/iGP. Lanes 2 and 6: MSPDBL2 DEL clone 1. Lanes 3 and 7: MSPDBL2 DEL clone 2. Lanes 4 and 8: MSPDBL2 DEL clone 3.

**Table S1.** Immunofluorescence microscopy counts of proportions of MSPDBL2 +ve schizonts among all those with at least 8 nuclei, for the 3D7/iGP parasite line in presence of 0 or 1 mM of Shield-1 reagent (GDV1 is overexpressed and stabilized in the presence of Shield-1). Also shown are the proportions of GDV1 +ve trophozoites. Four replicate experiments were performed.

| **Assay**  **Number** | **[Shield-1]**  **(µM)** | **GDV1 +ve/**  **DAPI +ve** | | | | **Proportion of GDV1+ trophozoites**  **(± 95%CI)** | | **MSPDBL2 +ve/**  **DAPI +ve** | | **Proportion of**  **MSPDBL2+ schizonts**  **(± 95%CI)** |
| --- | --- | --- | --- | --- | --- | --- | --- | --- | --- | --- |
| 1 |  |  | | |  | | |  | |  |
|  | 0 | 7/107 | | | **6.5** (3.0 – 13.1) | | | 4/694 | | **0.6** (0.2 – 1.5) |
|  | 1 | 85/102 | | | **83.3** (74.8 – 89.4)* | | | 289/612 | | **47.2** (43.3 – 51.2)* |
|  |  |  | | |  | | |  | |  |
| 2 | 0 | 4/310 | | | **1.3** (0.4 – 3.4) | | | 12/646 | **1.8** (1.0 – 3.3) | |
|  | 1 | 239/300 | | | **79.7** (74.7 – 83.8)* | | | 275/653 | **42.1** (38.4 – 45.9)* | |
|  |  |  | | | |  | |  |  | |
|  | 0 | 5/131 | | | | **3.8** (1.4 - 8.8) | | 9/1009 | **0.9** (0.4 – 1.7) | |
| 3 | 1 | 171/207 | | | | **82.6** (76.8 – 87.2)* | | 277/871 | **31.8** (28.8 – 34.9)* | |
|  |  |  | | | |  | |  |  | |
|  | 0 | 0/300 | | | | **0.0** | | 12/987 | **1.2** (0.7 – 2.1) | |
| 4 | 1 | 183/310 | | | | **59.03** (53.3-64.3)* | | 246/753 | **32.7** (29.4 – 36.1)* | |
|  |  |  | | | |  | |  |  | |
| **Mean** | **0** |  | | | | **2.9** | |  | **1.1** | |
|  | **1** |  | | | | **76.2** | |  | **38.5** | |
|  |  | |  |  | | |  |  |  |  |

**:*Fisher exact test p value: p ˂ 0.00001

**Table S2.** Immunofluorescence microscopy counts of proportions of MSPDBL2 +ve schizonts among all those with at least 8 nuclei, in the 3D7/iGP parasite line with varying concentrations of Shield-1 reagent (GDV1 is overexpressed and stabilized in the presence of Shield-1). Also shown are the gametocyte conversion rates (GCR) measured in the same cultures as the proportion of stage 1 gametocytes developing in the first cycle of re-invasion using anti-Pfs16 staining (GCR data from Stewart *et al*, *Microbiology Spectrum* 2022 <https://doi.org/10.1128/spectrum.02234-22>).

| **Assay**  **number** | **Concentration Shield-1** | **MSPDBL2**  **+ve** | **DAPI**  **+ve** | **Proportion of MSPDBL2+ schizonts**  **(95%CI)** | **Pfs16**  **+ve** | **DAPI**  **+ve** | **Pfs16 GCR**  **(95%CI) *** |
| --- | --- | --- | --- | --- | --- | --- | --- |
|  | 0 | 7 | 868 | **0.8**  (0.4 – 1.7) | 104 | 1171 | **8.9**  (7.4 – 10.6) |
|  | 0.055 | 310 | 1031 | **30.1**  (27.3 – 32.9) | 157 | 1104 | **14.2**  (12.3 – 16.4) |
| 1 | 0.15 | 163 | 595 | **27.4**  (24.0 – 31.1) | 188 | 1117 | **16.8**  (14.7 – 19.1) |
|  | 0.5 | 253 | 746 | **33.9**  (30.6 – 37.4) | 197 | 1131 | **17.4**  (15.3 – 19.7) |
|  | 1 | 383 | 1046 | **36.6**  (33.7 – 39.6) | 262 | 978 | **26.8**  (24.11 – 29.65) |
|  | 0 | 7 | 877 | **0.8**  (0.3 – 1.7) | 41 | 926 | **4.4**  (3.3 – 6.0) |
|  | 0.055 | 66 | 938 | **7.0**  (5.5 – 8.9) | 164 | 800 | **20.5**  (17.9 – 23.4) |
| 2 | 0.15 | 127 | 882 | **14.4**  (12.2 – 16.9) | 139 | 864 | **16.1**  (13.8 – 16.7) |
|  | 0.5 | 186 | 765 | **24.3**  (21.4 – 27.5) | 209 | 790 | **26.5**  (23.5 – 29.6) |
|  | 1 | 159 | 840 | **18.9**  (46.4 – 21.7) | 150 | 595 | **25.2**  (21.9 – 28.8) |
|  | 0 | 12 | 987 | **1.2**  (0.7 – 2.1) | 47 | 1043 | **4.5**  (3.4 – 5.9) |
|  | 0.055 | 77 | 922 | **8.3**  (6.7 – 10.3) | 70 | 788 | **8.8**  (7.1 – 11.1) |
| 3 | 0.15 | 150 | 849 | **17.7**  (15.2 – 20.4) | 140 | 629 | **22.3**  (19.2 – 25.7) |
|  | 0.5 | 193 | 806 | **23.9**  (21.1 – 27.0) | 273 | 730 | **37.4**  (34.0 – 41.0) |
|  | 1 | 246 | 753 | **32.7**  (29.4 – 36.1) | 267 | 701 | **38.1**  (34.6 – 41.7) |

**Table S3.** Immunofluorescence microscopy counts of proportions of MSPDBL2 +ve schizonts among all those with at least 8 nuclei, in the NF54 GDV1Δ39 and NF54 parasite lines. The proportion of MSPDBL2+ schizonts are shown together with the 95% CI.

| **Assay**  **number** | **Line** | **%**  **Parasitemia** | **MSPDBL2**  **+ve** | **DAPI**  **+ve** | **Proportion of MSPDBL2+**  **Schizonts (95%CI)** |  | **Fisher Exact test**  **(p value)** |
| --- | --- | --- | --- | --- | --- | --- | --- |
|  |  |  |  |  |  |  |  |
| 1 | NF54 | 3.0 | 13 | 986 | 1.3 (0.7-2.3) |  | 0.0002 |
|  | NF54 GDV1Δ39 | 1.8 | 0 | 1000 | 0 |  |  |
|  |  |  |  |  |  |  |  |
| 2 | NF54 | 8.8 | 6 | 993 | 0.6 (0.24-1.35) |  | 0.031 |
|  | NF54 GDV1Δ39 | 6.6 | 0 | 999 | 0 |  |  |
|  |  |  |  |  |  |  |  |
| 3 | NF54 | 4.0 | 12 | 988 | 1.2 (0.67-2.14) |  | 0.0005 |
|  | NF54 GDV1Δ39 | 6.5 | 0 | 1000 | 0 |  |  |
|  |  |  |  |  |  |  |  |
| **Mean** | **NF54** |  |  |  | **1.0** |  |  |
|  | **NF54 GDV1Δ39** |  |  |  | **0** |  |  |

**Table S4.** Immunofluorescence microscopy counts of proportions of MSPDBL2 +ve schizonts among all those with at least 8 nuclei, for 13 *P. falciparum* cultured lines with multiple culture replicates (74 preparations in total). The proportion of MSPDBL2+ schizonts are shown together with the 95% CI, and are matched with associated gametocyte conversion rate (GCR) data for 71 of the cultures (see footnote). For most of the replicates, the estimated parasitemia in the cycle during which the schizonts were tested for expression of MSPDBL2 is shown (% Para), as well as the next cycle parasitemia when the proportions of gametocytes were tested (% Para next cycle), and the fold change in parasitemia between these successive cycles (see footnote).

| **Line** | **Replicate** | **RBC**  **batch** | **%**  **Para** | **MSPDBL2**  **+ve** | **DAPI**  **+ve** | **Proportion of MSPDBL2+ schizonts (95% CI)** | **%**  **Para**  **next cycle** | **Fold**  **change** | **Pfs16 GCR**  **(95% CI)** |  |
| --- | --- | --- | --- | --- | --- | --- | --- | --- | --- | --- |
|  | 1 | 3 | - | 2 | 997 | 0.2 (0.0 - 0.8) | - | - | 0 |  |
| **F12** | 2 | 4 | - | 2 | 997 | 0.2 (0.0 – 0.8) | - | - | 0 |  |
|  | 3 | 10 | 1.3 | 8 | 990 | 0.8 (0.4 – 1.6) | 8.7 | 6.7 | 0 |  |
|  | 4 | 11 | 1.2 | 12 | 987 | 1.2 (0.7 – 2.1) | 5.1 | 4.3 | 0 |  |
|  | 5 | 11 | 1.3 | 11 | 413 | 2.6 (1.4 – 4.8) | 2.2 | 1.7 | 0 |  |
|  | ***Mean (SD)*** |  |  | ***0.9 (0.9)*** | | | |  | ***0*** |  |
|  |  |  |  |  | | | |  |  |  |
|  | 1 | 2 | - | 1 | 999 | 0.1 (0.0 – 0.6) | - |  | 0.1 (0 - 5.6) |  |
| **D10** | 2 | 2 | - | 4 | 1994 | 0.2 (0.1 – 0.5) | - |  | 0.8 (0.5 – 1.6) |  |
|  | 3 | 3 | - | 1 | 999 | 0.1 (0.0 – 0.6) | - |  | 0.2 (0.1 – 0.7) |  |
|  | 4 | 9 | 3.4 | 0 | 1000 | 0.0 (0.0 - 0.5) | 8.8 | 2.6 | 0.3 (0.1 – 0.9) |  |
|  | ***Mean (SD)*** |  |  | ***0.1 (0.1)*** | | | |  | ***0.35 (0.31)*** |  |
|  |  |  |  |  | | | |  |  |  |
|  | 1 | 13 | 1.1 | 0 | 999 | 0.0 (0.0 – 0.5) | 5.3 | 4.8 | 0.3 (0.1 – 1.0) |  |
|  | 2 | 13 | 1.5 | 8 | 991 | 0.8 (0.4 - 1.6) | 4.5 | 3.0 | 0.3 (0.1 – 0.9) |  |
| **T9/96** | 3 | 13 | 2.2 | 3 | 996 | 0.3 (0.1 - 0.9) | 7.6 | 3.5 | 0.3 (0.1 – 1.0) |  |
|  | 4 | 14 | 0.6 | 10 | 984 | 1.0 (0.5 - 1.9) | 12.5 | 21 | 0.2 (0.2 – 0.7) |  |
|  | 5 | 14 | 0.6 | 2 | 997 | 0.2 (0.0 - 0.8) | 7.8 | 13 | 0.7 (0.2 – 2.0) |  |
|  | ***Mean (SD)*** |  |  | ***0.5 (0.4)*** | | | |  | ***0.36 (0.19)*** |  |
|  |  |  |  |  | | | |  |  |  |
|  | 1 | 6 | 0.8 | 2 | 303 | 0.6 (0.0 – 2.5) | 1.0 | 1.3 | 1.2 (0.7 – 2.1) |  |
|  | 2 | 7 | 1.8 | 7 | 953 | 0.7 (0.3 – 1.5) | 2.7 | 1.5 | 2.7 (1.6 – 4.5) |  |
| **Palo Alto** | 3 | 7 | 1.7 | 1 | 998 | 0.1 (0.0 – 0.6) | 2.2 | 1.3 | 1.6 (0.7 – 3.6) |  |
|  | 4 | 9 | - | 4 | 944 | 0.4 (0.1 – 1.1) | - | - | 0.1 (0 – 0.6) |  |
|  | 5 | 9 | 1.4 | 0 | 383 | 0.0 (0.0 – 1.2) | 2.9 | 2.1 | 0.3 (0.1 – 0.9) |  |
|  | 6 | 9 | 0.6 | 1 | 332 | 0.3 (0.0 – 1.9) | 1.4 | 2.3 | 0.1 (0 – 0.6) |  |
|  | ***Mean (SD)*** |  |  | ***0.4 (0.3)*** | | | |  | ***1 (1.03)*** |  |
|  |  |  |  |  | | | |  |  |  |
|  | 1 | 2 | - | 0 | 329 | 0.0 (0.0 – 1.4) | - | - | 2.0 (1.3 – 3.1) |  |
|  | 2 | 3 | - | 1 | 999 | 0.1 (0.0 – 0.6) | - | - | 0.3 (0.1 – 0.9) |  |
|  | 3 | 3 | - | 2 | 997 | 0.2 (0.0 – 0.8) | - | - | 1.2 (0.7 – 2.1) |  |
| **3D7** | 4 | 4 | 0.8 | 2 | 1996 | 0.1 (0.0 - 0.4) | 2.6 | 3.3 | 2.0 (1.3 – 3.1) |  |
|  | 5 | 4 | - | 1 | 1080 | 0.1 (0.0 – 0.6) | - | - | 1.9 (1.2 – 3.0) |  |
|  | 6 | 12 | 0.9 | 1 | 422 | 0.2 (0.0 – 1.5) | 1.1 | 1.2 | 2.3 (1.5 – 3.5) |  |
|  | 7 | 12 | 1.1 | 8 | 860 | 0.9 (0.4 – 1.8) | 1.4 | 1.3 | 1.6 (1.0 – 2.6) |  |
|  | ***Mean (SD)*** |  |  | ***0.2 (0.3)*** | | | |  | ***1.6 (0.67)*** |  |

| **Line** | **Replicate** | **RBC**  **batch** | **%**  **Para.** | **MSPDBL2 +ve** | **DAPI**  **+ve** | **Proportion of**  **MSPDBL2+ Schizonts**  **(95% CI)** | **% Para.**  **next cycle** | **Fold**  **change** | | | | **Pfs16 GCR***  **(95% CI)** | | |  |
| --- | --- | --- | --- | --- | --- | --- | --- | --- | --- | --- | --- | --- | --- | --- | --- |
|  | 1 | 5 | - | 14 | 543 | 2.6 (1.5 – 4.3) | - | - | | | 9.0 (7.3 - 11.1) | | |  |  |
|  | 2 | 5 | - | 29 | 735 | 3.9 (2.7 – 5.6) | - | - | | | 1.6 (1.0 – 2,6) | | |  |  |
| **D6** | 3 | 5 | - | 26 | 974 | 2.6 (1.8 – 3.9) | - | - | | | 0.7 (0.3 – 1.5) | | |  |  |
|  | 4 | 9 | 1.0 | 2 | 997 | 0.2 (0.0 – 0.8) | 0.1 | 0.1 | | | 2.7 (1.8 – 3.9) | | |  |  |
|  | 5 | 9 | 1.4 | 7 | 647 | 1.1 (0.5 – 2.3) | 0.7 | 0.5 | | | 5.3 (4.0 – 6.9) | | |  |  |
|  | ***Mean (SD)*** |  |  |  |  | ***2.1 (1.4)*** |  |  | | ***3.8 (3.3)*** | | | |  |  |
|  |  |  |  |  | | | | |  | | |  |  | | |
|  | 1 | 6 | 1.4 | 28 | 972 | 2.8 (2.0 – 4.1) | 3.3 | 2.4 | | | | 2.2 (1.5 – 3.4) | | |  |
|  | 2 | 6 | 0.8 | 12 | 987 | 1.2 (0.7 – 2.1) | 1.5 | 1.9 | | | | 9.3 (7.6 – 11.4) | | |  |
|  | 3 | 6 | 2.5 | 36 | 729 | 4.9 (3.6 – 6.8) | 1.5 | 0.6 | | | | 10.0 (8.3 – 12.2) | | |  |
| **GB4** | 4 | 6 | 1.2 | 13 | 866 | 1.5 (0.8 – 2.6) | 1.0 | 0.8 | | | | 3.8 (2.8 – 5.3) | | |  |
|  | 5 | 7 | 0.9 | 35 | 823 | 4.2 (3.1 – 5.9) | 7.7 | 8.6 | | | | 6.3 (4.9 – 8.0) | | |  |
|  | 6 | 7 | 1.4 | 133 | 866 | 15.4 (13.1 – 17.9) | 3.6 | 2.6 | | | | 1.6 (1.0 – 2.6) | | |  |
|  | 7 | 9 | 0.9 | 172 | 829 | 20.7 (18.1 – 23.6) | 2.2 | 3.7 | | | | 3.5 (2.5 – 4.9) | | |  |
|  | ***Mean (SD)*** |  |  |  |  | ***7.3 (7.1)*** |  |  | | | | ***5.24 (3.36)*** | | |  |
|  |  |  |  |  |  |  |  |  | | | |  | | |  |
|  | 1 | 2 | 0.9 | 6 | 1995 | 0.3 (0.1 – 0.7) | 3.7 | 4.1 | | | | 14.4 (12.3 -16.9) | | |  |
|  | 2 | 3 | 1.5 | 1 | 95 | 1.0 (0.0 – 6.3) | 1.9 | 1.6 | | | | 0.4 (0.2 – 1.00 | | |  |
| **Dd2** | 3 | 4 | - | 1 | 104 | 1.0 (0.0 – 5.7) | - | - | | | | 4.8 (3.6 – 6.4) | | |  |
|  | 4 | 4 | - | 4 | 995 | 0.4 (0.1 – 1.1) | - | - | | | | 9.2 (7.5 – 11.2) | | |  |
|  | 5 | 12 | - | 0 | 380 | 0.0 (0.0 – 1.2) | - | - | | | | 4.3 (2.9 – 5.5) | | |  |
|  | 6 | 12 | - | 21 | 980 | 2.1 (1.4 – 3.3) | - | - | | | | 4.0 (2.9 – 5.5) | | |  |
|  | ***Mean (SD)*** |  |  |  |  | ***0.8 (0.7)*** |  |  | | | | ***6.1 (4.9)*** | | |  |
|  |  |  |  |  |  |  |  |  | | | |  | | |  |
|  | 1 | 2 | - | 69 | 467 | 14.8 (11.8 – 18.3) | - | - | | | | 7.2 (5.5 – 9.4) | | |  |
| **HB3** | 2 | 2 | - | 107 | 892 | 12.0 (10.0 – 14.3) | - | - | | | | 10.3 (8.6 – 12.3) | | |  |
|  | 3 | 9 | 3.4 | 119 | 880 | 13.5 (11.4 – 16.0) | 4.5 | 1.3 | | | | 6.1 (4.7 – 7.8) | | |  |
|  | 4 | 9 | 0.9 | 67 | 535 | 12.5 (10.0 – 15.6) | 7.5 | 8.3 | | | | 5.9 (4.6 – 7.6) | | |  |
|  | ***Mean (SD)*** |  |  |  |  | ***13.2 (1.2)*** |  |  | | | | ***7.3 (2.03)*** | | |  |
|  |  |  |  |  |  |  |  |  | | | |  | | |  |
|  | 1 | 2 | - | 17 | 983 | 1.7 (1.1 – 2.8) | - | - | | | | 22.0 (19.6 – 24.7) | | |  |
| **NF54** | 2 | 2 | - | 7 | 992 | 0.7 (0.3 – 1.5) | - | - | | | | 18.0 (15.7 –20.5) | | |  |
|  | 3 | 13 | - | 0 | 150 | 0.0 (0.0 – 3.0) | - | - | | | | 2.8 (1.9 – 4.0) | | |  |
|  | ***Mean (SD)*** |  |  |  |  | ***0.8 (0.8)*** |  |  | | | | ***14.2 (10.1)*** | | |  |

| **Line** | **Replicate** | **RBC**  **batch** | **%**  **Para.** | **MSPDBL2**  **+ve** | **DAPI**  **+ve** | **Proportion of MSPDBL2+**  **Schizonts**  **(95% CI)** | **%**  **Para.**  **next cycle** | **Fold**  **change** | **Pfs16**  **GCR ***  **(95% CI)** |  |
| --- | --- | --- | --- | --- | --- | --- | --- | --- | --- | --- |
|  | 1 | 5 | - | 2 | 340 | 0.6 (0.0 – 2.3) | - | - | 17.8 (15.4 – 20.4) |  |
|  | 2 | 5 | - | 1 | 494 | 0.2 (0.0 – 1.3) | - | - | 7.2 (5.7 – 9.0) | |
|  | 3 | 7 | 1.3 | 10 | 989 | 1.0 (0.5 – 1.9) | 1.1 | 0.8 | 7.5 (6.0 – 9.4) | |
| **RO33** | 4 | 7 | 2.4 | 0 | 422 | 0.0 (0.0 – 1.1) | 0.7 | 0.3 | 10.1 (8.3 – 12.2) | |
|  | 5 | 10 | - | 0 | 999 | 0.0 (0.0 – 0.5) | - | - | 7.8 (6.3 – 9.7) | |
|  | 6 | 10 | 1.4 | 2 | 349 | 0.6 (0.0 – 2.2) | 3.6 | 2.6 | 14.2 (12.0 – 16.6) | |
|  | ***Mean (SD)*** |  |  |  |  | ***0.4 (0.4)*** |  |  | ***10.7 (4.3)*** | |
|  |  |  |  |  |  |  |  |  |  | |
|  | 1 | 6 | 2.1 | 0 | 1000 | 0.0 (0.0 – 0.5) | 3.2 | 2.7 | 18.6 (16.1 – 21.3) |  |
|  | 2 | 6 | 1.2 | 0 | 474 | 0.0 (0.0 – 1.0) | 2.5 | 2.1 | 24.8 (22 – 27.9) |  |
| **7G8** | 3 | 10 | 1.9 | 3 | 332 | 0.9 (0.2 - 2.7) | 0.9 | 1.7 | 7.1 (4.8 – 10.3) |  |
|  | 4 | 10 | 2.2 | 2 | 545 | 0.3 (0.0 – 1.4) | 2.6 | 1.2 | 2.0 (1.0 -3.7) |  |
|  | 5 | 11 | 1.5 | 1 | 493 | 0.2 (0.0 – 1.3) | 2.2 | 1.5 | 4.8 (3.6 – 6.4) |  |
|  | ***Mean (SD)*** |  |  |  |  | ***0.3 (0.3)*** |  |  | ***11.5 (9.7)*** |  |
|  |  |  |  |  |  |  |  |  |  |  |
|  | 1 | 5 | 2.1 | 52 | 1257 | 4.1 (3.2 – 5.4) | 4.3 | 2.0 | 26.9 (23.9 – 30.1) |  |
|  | 2 | - | - | 18 | 1184 | 1.5 (0.9 – 2.4) | - | - | 23.5 (20.0 – 26.6) |  |
|  | 3 | - | - | 2 | 1336 | 0.1 (0.1 – 0.6) | - | - | 18.6 (16.1 – 21.4) |  |
| **FCC2** | 4 | 5 | 2.5 | 43 | 956 | 4.5 (3.3 – 6.0) | 3.9 | 1.6 | 17.6 (15.2 – 20.3) |  |
|  | 5 | 5 | 1.1 | 4 | 934 | 0.4 (0.1 – 1.1) | 4.8 | 4.4 | 3.3 (2.0 – 5.3) |  |
|  | 6 | 5 | 0.8 | 2 | 326 | 0.6 (0.0 – 2.4) | 3.0 | 3.8 | 4.6 (3.1 – 6.8) |  |
|  | 7 | 10 | - | 3 | 996 | 0.3 (0.1 – 1.0) | - | - | 7.4 (5.9 – 9.2) |  |
|  | 8 | 10 | - | 3 | 996 | 0.3 (0.1 – 0.9) | - | - | 4.9 (3.7 – 6.5) |  |
|  | ***Mean (SD)*** |  |  |  |  | ***1.5 (1.8)*** |  |  | **13.3 (9.3)** |  |
|  |  |  |  |  |  |  |  |  |  |  |

The GCR data for these cultures were reported previously (Stewart et al. 2022 *Microbiology Spectrum* <https://doi.org/10.1128/spectrum.02234-22>) and are aligned here to enable analysis.

The parasitemia estimates were made for culture maintenance and are not available for all replicates, but those available are included here for *post hoc* exploratory analysis (this shows no correlation between these variables and MSPDBL2 expression in schizonts).

**Table S5.** Mann Whitney pairwise comparisons of proportions of MSPDBL2+ schizonts in different *P. falciparum* cultured lines. Statistically significant comparisons are highlighted in green (P<0.05*, P<0.01**, P<0.0001***), analysing data in Supplementary Table S3. Significant comparisons carried out with NF54 (n=3) were non-significant with every line tested except for GB4 (P=0.048).

**Table S6.** Tests for correlation of parasitaemia with MSPDBL2 expression in different experimental replicate assays for different lab isolates. Spearman’s r (rho) correlation coefficients and P values are shown for each isolate, the % parasitaemia/MSPDBL2 expression are shown in table S4.

| Laboratory isolate | No. of assays | Correlation between parasitemia and MSPDBL2 expression in schizonts | | Correlation between parasitemia fold change and MSPDBL2 expression in schizonts |
| --- | --- | --- | --- | --- |
|  |  | **rho** | **P** | **rho P** |
| T9_96 | 5 | -0.05 | 1.00 | 0.1 0.95 |
| Palo Alto | 5 | 0.2 | 0.78 | -0.20 0.73 |
| GB4 | 7 | 0.32 | 0.51 | 0.42 0.35 |
| 7G8 | 5 | 0.36 | 0.56 | -0.67 0.26 |

**Table S7.** Effects of adding choline to serum-free medium on proportions of MSPDBL2+ schizonts in the HB3 and NF54 *P. falciparum* lines. Microscopical IFA counting of schizonts with at least 8 nuclei was performed on multiple experimental replicates of each line, with statistical analysis of proportions and effects within each replicate separately. The proportion of MSPDBL2+ schizonts are shown together with the 95% CI and are matched with associated gametocyte conversion rate (GCR) data measured in the same cultures as the proportion of stage 1 gametocytes developing in the first cycle of re-invasion using anti-Pfs16 staining (GCR data from Stewart et al. 2022 *Microbiology Spectrum* e0223422).

| **Replicate Assays** | **Condition** | **MSPDBL2 +ve** | **DAPI**  **+ve** | | **Proportion of MSPDBL2+ schizonts**  **(± 95%CI)** | | **Fisher’s Exact Test** | | **Pfs16**  **+ve**  **(GCR)*** | | **Pfs16**  **-ve**  **(GCR)*** | | **Pfs16**  **GCR *** | | | **Fisher’s Exact Test**  **(p value)** |
| --- | --- | --- | --- | --- | --- | --- | --- | --- | --- | --- | --- | --- | --- | --- | --- | --- |
|  |  | **HB3** | |  | |  | |  | |  | |  | |  |  |  |
| R1 | - choline | 100 | 842 | | 11.8 (9.8 – 14.2) | | 0.0004 | | 16 | | 967 | | 1.6 (1.0 – 2.6) | | | 0.141 |
|  | + choline | 65 | 934 | | 6.9 (5.5 – 8.8) | |  | | 7 | | 864 | | 0.8 (0.4 – 1.6) | | |  |
|  |  |  |  | |  | |  | |  | |  | |  | | |  |
| R2 | - choline | 103 | 902 | | 11.4 (9.5 -13.7) | | < 0.00001 | | 34 | | 902 | | 3.6 (2.6 – 5.0) | | | < 0.00001 |
|  | + choline | 46 | 953 | | 4.8 (3.6 – 6.4) | |  | | 3 | | 993 | | 0.3 (0.1 – 0.9) | | |  |
|  |  |  |  | |  | |  | |  | |  | |  | | |  |
| R3 | - choline | 128 | 881 | | 14.5 (12.3 – 17.0) | | < 0.00001 | | 36 | | 929 | | 3.7 (2.7 – 5.1) | | | 0.013 |
|  | + choline | 63 | 976 | | 6.4 (5.1 – 8.2) | |  | | 18 | | 950 | | 1.9 (1.2 – 2.9) | | |  |
|  |  |  |  | |  | |  | |  | |  | |  | | |  |
| R4 | - choline | 172 | 1120 | | 15.3 (13.3 – 17.6) | | < 0.00001 | | 24 | | 954 | | 2.5 (1.7 – 3.6) | | | 0.446 |
|  | + choline | 63 | 930 | | 6.8 (5.3 – 8.6) | |  | | 19 | | 961 | | 1.9 (1.2 – 3.0) | | |  |
|  |  |  |  | |  | |  | |  | |  | |  | | |  |
| R5 | - choline | 34 | 758 | | 4.5 (3.2 – 6.2) | | 0.900 | | 20 | | 959 | | 2.0 (1.3 – 3.1) | | | 0.8763 |
|  | + choline | 32 | 739 | | 4.3 (3.1 – 6.1) | |  | | 21 | | 957 | | 2.1 (1.4 – 3.3) | | |  |
|  |  |  |  | |  | |  | |  | |  | |  | | |  |
| R6 | - choline | 60 | 939 | | 6.4 (5.0 - 8.1) | | 0.055 | | 19 | | 962 | | 1.9 (1.2 – 3.0) | | | 0.0323 |
|  | + choline | 81 | 925 | | 8.7 (7.1 - 10.7) | |  | | 8 | | 983 | | 0.8 (0.4 – 1.6) | | |  |
|  |  |  |  | |  | |  | |  | |  | |  | | |  |
| R7 | - choline | 76 | 923 | | 8.2 (6.6 – 10.1) | | 0.223 | | 29 | | 943 | | 3.0 (2.1 – 4.3) | | | 0.0004 |
|  | +choline | 90 | 909 | | 9.9 (8.1 – 12) | |  | | 8 | | 983 | | 0.8 (0.4 – 1.6) | | |  |
|  |  |  |  | |  | |  | |  | |  | |  | | |  |
| **Mean** | **– choline** |  |  | | **12** | |  | |  | |  | | **2.6** | | |  |
|  | **+ choline** |  |  | | **7.9** | |  | |  | |  | | **1.2** | | |  |

| **Replicate Assays** | **Condition** | **MSPDBL2 +ve** | **DAPI**  **+ve** | **Proportion of PfMSPDBL2+ schizonts**  **(± 95%CI)** | **Fisher’s**  **Exact Test** | **Pfs16**  **+ve**  **(GCR)*** | **Pfs16**  **-ve**  **(GCR)*** | **Pfs16 GCR*** | **Fisher’s Exact Test**  **(p value)** |  | |
| --- | --- | --- | --- | --- | --- | --- | --- | --- | --- | --- | --- |
|  |  | **NF54** | |  |  |  |  |  |  | |  |
| R1 | - choline | 2 | 577 | 0.3 (0.0 – 1.3) | 0.062 | 27 | 945 | 2.8 (1.9 – 4.0) | 0.0083 |  | |
|  | + choline | 13 | 962 | 1.3 (0.7 - 2.3) |  | 11 | 978 | 1.1 (0.6 – 2.0) |  |  |  |
|  |  |  |  |  |  |  |  |  |  |  |  |
| R2 | - choline | 2 | 997 | 0.2 (0.0 – 0.8) | 0.272 | 30 | 943 | 3.1 (2.2 – 4.4) | 0 |  |  |
|  | + choline | 2 | 343 | 0.6 (0.0 – 2.2) |  | 6 | 990 | 0.6 (0.3 – 1.3) |  |  |  |
|  |  |  |  |  |  |  |  |  |  |  |  |
| R3 | - choline | 2 | 776 | 0.2 (0.0 – 1.0) | 1.0 | 40 | 819 | 4.7 (3.4 – 6.3) | 0 |  |  |
|  | + choline | 3 | 996 | 0.3 (0.0 – 0.9) |  | 13 | 973 | 1.3 (0.8 – 2.2) |  |  |  |
|  |  |  |  |  |  |  |  |  |  |  |  |
| R4 | - choline | 6 | 984 | 0.6 (0.2 – 1.3) | 0.178 | 43 | 913 | 4.5 (3.4 – 6.0) | < 0.00001 |  |  |
|  | + choline | 2 | 988 | 0.2 (0.0 – 0.8) |  | 8 | 983 | 0.8 (0.4 – 1.6) |  |  |  |
|  |  |  |  |  |  |  |  |  |  |  |  |
| R5 | - choline | 9 | 973 | 0.9 (0.4 – 1.8) | 0.628 | 70 | 861 | 7.5 (6.0 – 9.4) | 0.0219 |  |  |
|  | + choline | 4 | 973 | 0.4 (0.1 - 1.1) |  | 47 | 905 | 4.9 (3.7 – 6.5) |  |  |  |
|  |  |  |  |  |  |  |  |  |  |  |  |
| R6 | - choline | 0 | 863 | 0.0 (0.0 – 0.5) | N/A | 8 | 983 | 0.8 (0.4-1.6) | 0.5798 |  |  |
|  | + choline | 0 | 640 | 0.0 (0.0 – 0.7) |  | 5 | 986 | 0.5 (0.2 – 1.2) |  |  |  |
|  |  |  |  |  |  |  |  |  |  |  |  |
| **Mean** | **- choline** |  |  | **0.4** |  |  |  | **3.9** |  |  |  |
|  | **+ choline** |  |  | **0.5** |  |  |  | **1.5** |  |  |  |

**Table S8.** Immunofluorescence microscopy counts of proportions of MSPDBL2 +ve schizonts in genetically-engineered parasite lines (among all those with at least 8 nuclei), using α-MSPDBL2 antibodies raised against conserved sequence N-terminal (Nter) or C-terminal (Cter) MSPDBL2 recombinant proteins. The tested lines are: the MSPDBL2 tagged line (MSPDBL2-TAG) and the MSPDBL2 disrupted line (MSPDBL2 DEL). Replicates and clones are indicated in brackets. Schizonts here are examined immediately after parasite cultures had been treated with G418 for a week to ensure selection of the engineered expression, and high proportions of schizonts expressingthe engineered MSPDBL2 are maintained for at least 6 days after removal of the G418 treatment (as shown separately in Supplementary Table S9).

| **Assay number** | **Line (and antibody)** | **MSPDBL2**  **+ve** | **DAPI**  **+ve** | **Proportion of MSPDBL2+ schizonts**  **(95% CI)** |
| --- | --- | --- | --- | --- |
|  | MSPDBL2-TAG rep1 (Nter) | 331 | 508 | 65.2 (60.9 – 69.2) |
|  | MSPDBL2-TAG rep1 (Cter) | 232 | 368 | 63.0 (58.0 – 67.8) |
|  | MSPDBL2-TAG rep2 (Nter) | 233 | 341 | 68.3 (63.2 – 73.0) |
| **1** | MSPDBL2-TAG rep2 (Cter) | 145 | 183 | 79.2 (72.8 – 84.5) |
|  |  |  |  |  |
|  | MSPDBL2 DEL Cl1 (Nter) | 480 | 520 | 92.3 (89.7-94.3) |
|  | MSPDBL2 DEL Cl1 (Cter) | 0 | 930 | 0.0 |
|  | MSPDBL2 DEL Cl3 (Nter) | 373 | 403 | 92.6 (89.5-94.7) |
|  | MSPDBL2 DEL Cl3 (Cter) | 0 | 570 | 0.0 |
|  |  |  |  |  |
|  |  |  |  |  |
|  | MSPDBL2-TAG rep1 (Nter) | 261 | 309 | 84.5 (80.0-88.1) |
|  | MSPDBL2-TAG rep1 (Cter) | 246 | 314 | 78.3 (73.4-82.6) |
| **2** |  |  |  |  |
|  | MSPDBL2 DEL Cl1 (Nter) | 448 | 551 | 81.3 (77.8-84.3) |
|  | MSPDBL2 DEL Cl1 (Cter) | 0 | 640 | 0.0 |
|  |  |  |  |  |
|  |  |  |  |  |
|  | **MSPDBL2-TAG (Nter)** |  |  | **72.6** |
|  | **MSPDBL2-TAG (Cter)** |  |  | **73.5** |
| **Mean** |  |  |  |  |
|  | **MSPDBL2 DEL (Nter)** |  |  | **88.7** |
|  | **MSPDBL2 DEL (Cter)** |  |  | **0** |
|  |  |  |  |  |
|  |  |  |  |  |

**Table S9.** Engineered parasite lines MSPDBL2-TAG and MSPDBL2 DEL continue to respectively express intact or truncated MSPDBL2 in a majority of mature schizonts after six days following removal of the G418 selection. IFA microscopy counts are shown of proportions of schizonts in (among all those with at least 8 nuclei) expressing the N-terminal portion of MSPDBL2 using antibodies raised against a conserved sequence N-terminal MSPDBL2 recombinant protein. Three different replicate cultures were assayed for each of the MSPDBL2 tagged line (MSPDBL2-TAG) and three clones of the MSPDBL2 disrupted line (MSPDBL2 DEL). The proportions of MSPDBL2 +ve schizonts are similar as those in the final cycle when G418 selection is present (Figure 6 and Supplementary Table S8).

| **Assay number** | **Line** | **DBL2**  **+ve** | **DAPI**  **+ve** | **Proportion of MSPDBL2+ schizonts**  **(95% CI)** |
| --- | --- | --- | --- | --- |
| IFA assays performed on mature schizonts harvested three cycles after the end of the G418 treatment | | | | |
|  |  |  |  |  |
|  | MSPDBL2-TAG (rep1) | 252 | 337 | 74.8 (69.8-79.3) |
| 1 | MSPDBL2 DEL (Cl1) | 229 | 308 | 74.35 (69.1-79.1) |
|  | MSPDBL2 DEL (Cl2) | 268 | 383 | 70.0 (65.1-74.5) |
|  | MSPDBL2 DEL (Cl3) | 248 | 297 | 83.5 (78.8-87.5) |
|  |  |  |  |  |
|  | MSPDBL2-TAG (rep2) | 256 | 310 | 82.6 (77.9-86.6) |
| 2 | MSPDBL2 DEL (Cl1) | 282 | 307 | 91.9 (88.2-94.7) |
|  | MSPDBL2 DEL (Cl2) | 242 | 301 | 80.4 (75.5-84.7) |
|  | MSPDBL2 DEL (Cl3) | 289 | 394 | 73.3 (68.7-77.6) |
|  |  |  |  |  |
|  | MSPDBL2-TAG (rep3) | 261 | 271 | 96.3 (93.3-98.2) |
| 3 | MSPDBL2 DEL (Cl1) | 464 | 555 | 83.6 (80.3-86.6) |
|  | MSPDBL2 DEL (Cl2) | 242 | 334 | 72.5 (67.3-77.2) |
|  | MSPDBL2 DEL (Cl3) | 260 | 287 | 90.6 (86.6-93.7) |
|  |  |  |  |  |
|  |  |  |  |  |

**Table S10.** Gametocyte conversion rates of genetically engineered lines with multiple experimental replicates in the presence and absence of Shield-1 reagent. The tested lines are: 3D7/iGP (parental line), the MSPDBL2 tagged line (MSPDBL2-TAG) and the MSPDBL2 disrupted line (MSPDBL2 DEL). Assays were performed after removal of G418 selection on the MSPDBL2-engineered lines so that the parental 3D7/iGP line can also be compared.

| **Assay number** | **Line (and treatment)** | | **Pfs16**  **+ve** | **DAPI**  **+ve** | | **Pfs16 GCR**  **(95% CI)** | |  |
| --- | --- | --- | --- | --- | --- | --- | --- | --- |
|  |  | |  |  | |  | |  |
|  | 3D7/iGP rep1 (- Shield-1) | | 47 | 992 | | 4.7 (3.6 –6.2) | |  |
|  | 3D7/iGP rep1 (+ Shield-1) | | 349 | 690 | | 50.6 (46.9 – 54.3) | |  |
| 1 | MSPDBL2-TAG rep1 (- Shield-1) | | 104 | 791 | | 13.1 (11.0 – 15.7) | |  |
|  | MSPDBL2-TAG rep1 (+ Shield-1) | | 310 | 676 | | 45.9 (42.1 –49.6) | |  |
|  | MSPDBL2 DEL Cl1 (- Shield-1) | | 62 | 937 | | 6.6 (5.2-8.4) | |  |
|  | MSPDBL2 DEL Cl1 (+ Shield-1) | | 349 | 670 | | 52.1 (48.3-55.8) | |  |
|  |  | |  |  | |  | |  |
|  | 3D7/iGP rep 2 (- Shield-1) | | 39 | 929 | | 4.2 (3.1 – 5.7) | |  |
|  | 3D7/iGP rep 2 (+ Shield-1) | | 302 | 507 | | 59.6 (55.2-63.7) | |  |
| 2 | MSPDBL2-TAG rep2 (- Shield-1) | | 81 | 806 | | 10.0 (8.1–12.3) | |  |
|  | MSPDBL2-TAG rep2 (+ Shield-1) | | 402 | 837 | | 48.0 (44.7–51.4) | |  |
|  | MSPDBL2 DEL Cl2 (- Shield-1) | | 88 | 913 | | 9.6 (7.9-11.7) | |  |
|  | MSPDBL2 DEL Cl2 (+ Shield-1) | | 289 | 670 | | 43.1 (39.4-46.9) | |  |
|  |  | |  |  | |  | |  |
|  | 3D7/iGP rep 3 (- Shield-1) | | 93 | 849 | | 10.9 (9.0-13.2) | |  |
|  | 3D7/iGP rep 3 (+ Shield-1) | | 193 | 568 | | 34.0 (30.2-38.0) | |  |
| 3 | MSPDBL2-TAG rep3 (- Shield-1) | | 73 | 903 | | 8.1 (6.5-10.0) | |  |
|  | MSPDBL2-TAG rep3 (+ Shield-1) | | 269 | 758 | | 35.5 (32.2-39.0) | |  |
|  | MSPDBL2 DEL Cl3 (- Shield-1) | | 66 | 901 | | 7.3 (5.8-9.2) | |  |
|  | MSPDBL2 DEL Cl3 (+ Shield-1) | | 249 | 775 | | 32.13 (29.0-35.5) | |  |
|  |  | |  |  | |  | |  |
|  | 3D7/iGP rep1 (- Shield-1) | | 61 | 938 | | 6.5 (5.1-8.3) | |  |
|  | 3D7/iGP rep1 (+ Shield-1) | | 367 | 662 | | 55.4 (51.6-59.2) | |  |
| 4 | MSPDBL2-TAG rep1 (- Shield-1) | | 43 | 956 | | 4.5 (3.3 - 6.0) | |  |
|  | MSPDBL2-TAG rep1 (+ Shield-1) | | 358 | 651 | | 55.0 (51.1 - 58.8) | |  |
|  | MSPDBL2 DEL Cl1 (- Shield-1) | | 60 | 942 | | 6.4 (5.0 - 8.1) | |  |
|  | MSPDBL2 DEL Cl1 (+ Shield-1) | | 330 | 626 | | 52.7 (48.8 - 56.6) | |  |
|  |  | |  |  | |  | |  |
|  | 3D7/iGP rep 2 (- Shield-1) | | 77 | 931 | | 8.3 (6.7-10.2) | |  |
|  | 3D7/iGP rep 2 (+ Shield-1) | | 383 | 643 | | 59.6 (55.7-63.3) | |  |
| 5 | MSPDBL2-TAG rep2 (- Shield-1) | | 66 | 935 | | 7.1 (5.6- 8.9) | |  |
|  | MSPDBL2-TAG rep2 (+ Shield-1) | | 366 | 639 | | 57.3 (53.4-61.1) | |  |
|  | MSPDBL2 DEL Cl2 (- Shield-1) | | 44 | 583 | | 7.5 (5.6 - 10.0) | |  |
|  | MSPDBL2 DEL Cl2 (+ Shield-1) | | 5 | 12 | | 41.7 (19.3 -68.1) | |  |
|  |  | |  |  | |  | |  |
|  | 3D7/iGP rep 3 (- Shield-1) | | 80 | 900 | | 8.9 (7.2-10.9) | |  |
|  | 3D7/iGP rep 3 (+ Shield-1) | | 336 | 703 | | 47.8 (44.1 - 51.5) | |  |
| 6 | MSPDBL2-TAG rep3 (- Shield-1) | | 47 | 951 | | 5.0 (3.7 - 6.5) | |  |
|  | MSPDBL2-TAG rep3 (+ Shield-1) | | 315 | 627 | | 50.2 (46.3 - 54.1) | |  |
|  | MSPDBL2 DEL Cl3 (- Shield-1) | | 70 | 875 | | 8.0 (6.4 - 10.0) | |  |
|  | MSPDBL2 DEL Cl3 (+ Shield-1) | | 365 | 641 | | 56.9 (53.1- 60.7) | |  |
|  |  | |  |  | |  | |  |
|  | **3D7/iGP (- Shield-1)** |  | | |  | | **7.2** | |
|  | **3D7/iGP (+ Shield-1)** |  | | |  | | **51.1** | |
|  | **MSPDBL2-TAG (- Shield-1)** |  | | |  | | **7.9** | |
| **Mean** | **MSPDBL2-TAG (+ Shield-1)** |  | | |  | | **48.6** | |
|  | **MSPDBL2 DEL (- Shield-1)** |  | | |  | | **7.6** | |
|  | **MSPDBL2 DEL (+ Shield-1)** |  | | |  | | **47.4** | |
|  |  |  | | |  | |  | |

**Table S11.** Multiplication rates of genetically-engineered *P. falciparum* lines in 6-day assays of exponential growth. Parasite genome copy numbers (Log_10_) were obtained by qPCR analysis using assay conditions as described in a previously published method (Murray et al. 2017, Scientific Reports 7:6436). Multiplication rates were calculated using data from all three replicates for each line. Assays were performed after removal of G418 selection on the MSPDBL2-engineered lines so that the parental 3D7 and 3D7/iGP lines can also be compared. Separate assays have shown that the majority of schizonts in the engineered lines normally still express the engineered MSPDBL2 after 6 days of culture without G418 (Supplementary Table S9).

|  | **Genome copy numbers (Log_10_) in 1µl DNA from each of three replicate growth assays** | | | |  |
| --- | --- | --- | --- | --- | --- |
| **Parasite Line** | **Log qPCR**  **Day 0** | **Log qPCR**  **Day 2** | **Log qPCR**  **Day 4** | **Log qPCR**  **Day 6** | **Multiplication rate (95% CI) per 48h** |
|  |  |  |  |  |  |
| 3D7 | 2.71  2.87  2.85 | 3.96  4.00  3.96 | 4.66  4.65  4.75 | 5.31  5.65  5.37 | 7.26 (5.89-8.96) |
|  |  |  |  |  |  |
| 3D7/iGP | 1.86  1.65  1.89 | 2.84  2.73  2.87 | 3.26  3.67  3.36 | 4.15  4.52  4.28 | 6.53 (5.22-8.17) |
| MSPDBL2-TAG (rep1) | 2.37  2.45  2.57 | 3.79  3.59  3.69 | 4.70  4.82  5.33 | 5.16  5.33  5.28 | 8.81 (6.61-11.76) |
| MSPDBL2-TAG (rep2) | 2.83  2.60  2.67 | 3.59  3.91  3.74 | 4.60  4.89  4.56 | 5.21  5.62  5.28 | 7.91 (6.24-10.02) |
|  |  |  |  |  |  |
| MSPDBL2-TAG (rep3) | 2.50  2.57  2.51 | 3.73  3.63  3.65 | 4.43  4.50  4.48 | 4.99  5.25  5.28 | 7.46 (6.14-9.08) |
|  |  |  |  |  |  |
| MSPDBL2 DEL (clone 1) | 2.24  2.02  2.27 | 3.22  3.33  2.64 | 4.05  4.33  4.14 | 4.86  5.08  4.94 | 8.82 (6.70-11.60) |
| MSPDBL2 DEL (clone 2) | 2.14  2.12  2.22 | 3.23  3.29  3.32 | 3.80  4.18  3.85 | 4.86  4.98  4.93 | 6.48 (5.19-8.08) |
| MSPDBL2 DEL (clone 3) | 2.27  2.43  2.54 | 3.29  3.45  3.37 | 4.12  4.16  4.35 | 4.56  4.97  4.99 | 7.84 (6.46-9.50) |
|  |  |  |  |  |  |

**Table S12.** Sequences of primers designed for cloning and genotyping in the present study.

**Table S13.** *mspdbl2-tag* and *mspdbl2 del* sequences inserted into the endogenous locus:

*A. mspdbl2-TdTom-3xHA* sequence (3867 bp)*:*

ATGATATATATTTTATCTATTGTATTTTATATATTTTTTTTACATATTGATATATATGTAAACATTTATTCTACATGTTTTGTTGTAAATGAGGGGAACCCTAATTTAAGAAATAACATAATTAATGATGATGAACTAAAGGGGAAAGCATATAATAATACTATAGATGCTAATAACCAAAATATAGAATATAATAAAAACTTAAAGCACAATGTAAACTCATCTCATATATCTAAATTTTCGGATATTATGGATCAAGAAGATAAAGGAGATAATGAAAATTCTCATGACATAAAATTTGAAGAAAAAAAAAATATTAATAAATCTTTAGACGCTGAATCCAATTATGGTATTAATGAAATTAGTATTACTGGTAATGATAGTAATAGTGATAATAGTAATCAGAATATTTTTCCAGATGGTAGTGAATTAGCTGGAGGTATTCCTCGTTCTATATATACTATTAACCTTGGTTTTAATAAATGTCCTACTGAAGAGATTTGTAAAGACTTTAGTAATCTTCCACAATGTCGAAAGAATGTACATGAAAGAAATAATTGGTTGGGCTCAAGTGTAAAAAATTTTTCAAGTGATAATAAGGGGGTTCTTGTTCCTCCAAGAAGACAATCTTTATGTTTAAGAATTACATTACAAGATTTTCGTACGAAAAAGAAAAAGGAAGGAGATTTTGAAAAATTTATTTATTCATATGCATCATCTGAAGCTAGAAAATTAAGAACCATACACAATAATAACTTAGAAAAAGCTCATCAAGCTATAAGATATAGTTTTGCAGATATTGGAAATATTATTAGAGGAGATGACATGATGGATACACCTACGTCAAAAGAAACCATAACATATTTAGAAAAAGTACTTAAAATTTATAATGAAAATAATGATAAACCAAAAGATGCAAAAAAATGGTGGACAGAAAACAGGCATCATGTTTGGGAAGCAATGATGTGCGGATATCAGAGTGCGCAGAAAGATAACCAATGTACAGGTTATGGTAACATTGATGATATACCACAATTTTTAAGGTGGTTCAGAGAGTGGGGAACATATGTCTGTGAAGAAAGCGAAAAAAATATGAACACACTAAAAGCTGTTTGCTTTCCGAAACAGCCAAGAACCGAAGCGAATCCTGCATTGACTGTACATGAAAATGAAATGTGCTCATCAACTTTAAAAAAATATGAAGAATGGTATAATAAAAGGAAAACTGAATGGACTGAACAATCTATTAAATATAACAATGACAAAATTAATTATACAGATATAAAAACATTATCTCCTTCTGAATATTTAATAGAAAAATGTCCTGAATGTAAATGTACCAAAAAAAATTTGCAAGATGTATTTGAACTTACATTTGATGGAAAAGCTTTATTAGAAAAGCTAAAAAAAGAAGAATCACCTGTGAGTAATAGTGTGAATGCCTTACCTGAACCAGGTCAAATTACATTACCTGATCCTTCATTAAAACAAACAACACAACAGGAAAATCAACCTGTTGTAGAAACACCTGTTACCACAGCTGTTATTAATGAACATCAAGGACAAACAGAACCGAATAAAGGTGACAACAATAATGAAAGAGAAAATCATGAAAGTAATGTTGGTAGCATCCAAGAAGTAAACCAAGGTAGCGTGAGCGAAGAATCACATTCTAAAACTATAGATCCTTCTAAGATTGACGACCGTTTGGAATTAAGTAGTGGGTCATCATCTCTTGAACAACACTCTAAGGAAGATGTAAAAAAGGGATGTGCTTTAGAATTGGTACCTTTATCTTTATCGGATATTGAACAGATAGCTAATGAAAGCGAAGATGTACTGGAAGAGATAGAAGAAGAAATTAATACAGATGGGGAAATAGAATATATAACAGAAGAAGAAATAAAAGAAGATATAGAAGAAGAAACAGAAGAAGATATAGAAGAAGAAACAGAAGAAGAAACAGAAGAAGAAACAGAAGAAGAAGCAGATGAAGAAACAGTAAAAGAAATAGAAGACAAACCAGAACAAGAAATTAAAAATAAATCGCTAGAAGAAAAACAAATAGATAAAAATACAGATACCAGTGAAAAGAAAGGATTTAATAATTCAGAAAAAGATGAAAAAGCTCGAAATTTAATTTCTAAAAATTATAAAAATTATAATGAACTAGATAAAAACGTTCATACTTTAGTAAATTCAATTATTAGTTTATTAGAAGAAGGTAATGGAAGTGATTCTACCTTGAATAGTTTATCAAAAGATATTACAAATTTATTTAAAAATGGATCCGTGAGCAAGGGCGAGGAGGTCATCAAAGAGTTCATGCGCTTCAAGGTGCGCATGGAGGGCTCCATGAACGGCCACGAGTTCGAGATCGAGGGCGAGGGCGAGGGCCGCCCCTACGAGGGCACCCAGACCGCCAAGCTGAAGGTGACCAAGGGCGGCCCCCTGCCCTTCGCCTGGGACATCCTGTCCCCCCAGTTCATGTACGGCTCCAAGGCGTACGTGAAGCACCCCGCCGACATCCCCGATTACAAGAAGCTGTCCTTCCCCGAGGGCTTCAAGTGGGAGCGCGTGATGAACTTCGAGGACGGCGGTCTGGTGACCGTGACCCAGGACTCCTCCCTGCAGGACGGCACGCTGATCTACAAGGTGAAGATGCGCGGCACCAACTTCCCCCCCGACGGCCCCGTAATGCAGAAGAAGACCATGGGCTGGGAGGCCTCCACCGAGCGCCTGTACCCCCGCGACGGCGTGCTGAAGGGCGAGATCCACCAGGCCCTGAAGCTGAAGGACGGCGgccactacctggtggagttcaagACCATCTACATGGCCAAGAAGCCCGTGCAACTGCCCGGCTACTACTACGTGGACACCAAGCTGGACATCACCTCCCACAACGAGGACTACACCATCGTGGAACAGTACGAGCGCTCCGAGGGCCGCCACCACCTGTTCCTGGGGCATGGCACCGGCAGCACCGGCAGCGGCAGCTCCGGCACCGCCTCCTCCGAGGACAACAACATGGCCGTCATAAAAGAATTTATGAGATTCAAGGTACGTATGGAAGGTTCTATGAATGGACATGAGTTTGAGATTGAAGGAGAAGGTGAAGGTCGTCCTTATGAGGGAACCCAAACTGCAAAACTTAAGGTAACTAAAGGTGGTCCATTGCCATTTGCTTGGGATATATTGAGTCCACAATTCATGTACGGTAGTAAGGCCTACGTAAAACATCCAGCCGATATTCCAGATTACAAAAAGCTTTCATTCCCTGAGGGATTCAAGTGGGAAAGAGTTATGAATTTCGAAGATGGTGGTCTTGTAACCGTAACCCAAGATTCTTCACTTCAAGATGGAACTTTAATATATAAAGTAAAAATGAGAGGAACCAATTTCCCTCCTGATGGACCTGTAATGCAAAAAAAAACAATGGGTTGGGAGGCAAGTACAGAGAGATTGTACCCACGTGATGGTGTTCTTAAGGGAGAGATCCATCAAGCTTTGAAATTGAAGGATGGTGGACATTACTTGGTAGAGTTTAAGACTATATATATGGCAAAGAAGCCAGTTCAATTGCCTGGTTATTACTACGTTGATACTAAGTTAGATATTACAAGTCATAATGAGGATTATACCATAGTAGAACAATATGAACGTAGTGAAGGAAGACACCATTTGTTTCTGTACGGCATGGACGAGCTGTACAAGggtaccTCTGCTTGGAGTCATCCTCAATTTGAAAAAGGAGGATCTAGTTACCCTTACGATGTTCCTGACTATGCGGGCTATCCCTATGACGTCCCGGACTATGCCATGGGCTACCCTTACGACGTTCCAGATTACGCTGGAGGTTCTGGT

Sequence inserted into pL2M2_TdTom_T2A/G418 (BamH1/Td-TOM/ Kpn1/3xHA tag)

Predicted MW of translated product: 147.2 KDa

*B. mspdbl2 del* sequence *(681 bp):*

ATGATATATATTTTATCTATTGTATTTTATATATTTTTTTTACATATTGATATATATGTAAACATTTATTCTACATGTTTTGTTGTAAATGAGGGGAACCCTAATTTAAGAAATAACATAATTAATGATGATGAACTAAAGGGGAAAGCATATAATAATACTATAGATGCTAATAACCAAAATATAGAATATAATAAAAACTTAAAGCACAATGTAAACTCATCTCATATATCTAAATTTTCGGATATTATGGATCAAGAAGATAAAGGAGATAATGAAAATTCTCATGACATAAAATTTGAAGAAAAAAAAAATATTAATAAATCTTTAGACGCTGAATCCAATTATGGTATTAATGAAATTAGTATTACTGGTAATGATAGTAATAGTGATAATAGTAATCAGAATATTTTTCCAGATGGTAGTGAATTAGCTGGAGGTATTCCTCGTTCTATATATACTATTAACCTTGGTTTTAATAAATGTCCTACTGAAGAGATTTGTggtaccTCTGCTTGGAGTCATCCTCAATTTGAAAAAGGAGGATCTAGTTACCCTTACGATGTTCCTGACTATGCGGGCTATCCCTATGACGTCCCGGACTATGCCATGGGCTACCCTTACGACGTTCCAGATTACGCTGGAGGTTCTGGT

*mspdbl2* sequence inserted into pL2M2_T2A/G418 (Kpn1/3xHA tag)

Predicted MW of translated product: 24.5 KDa
